# Plant Diversity of Mts. Oligirtos and Farmakas (NE Peloponnisos, Greece) with Emphasis on Their Endemic Flora

**DOI:** 10.3390/plants11192649

**Published:** 2022-10-09

**Authors:** Andreas Zikos, Theophanis Constantinidis

**Affiliations:** Section of Ecology and Systematics, Department of Biology, National and Kapodistrian University of Athens, Panepistimiopolis, 15784 Athens, Greece

**Keywords:** new floristic records, endemic plants, habitats, IUCN categories, mountain flora, threats

## Abstract

Greece is known to be a biodiversity hotspot. Though the plant diversity of Peloponnisos, the southernmost part of the Greek mainland, has been well-studied during the past 200 years, there are still gaps in our knowledge. To this end, the flora of the neighboring mountains Oligirtos and Farmakas was investigated, with a total of 740 and 762 taxa (species and subspecies) recorded, respectively, of which 635 and 756 for the first time. Ten species or subspecies were previously not known from Peloponnisos. Endemics correspond to 10.2% and 8.9% of the total flora and are predominately hemicryptophytes and entomogamous. Almost half of them produce capsules. The number of endemics per 2 × 2 km grid cell reveals that their highest number is found in areas of high elevation, and corresponds to habitats above the tree line, or to the limestone cliffs vegetation. No less than 62 endemic plant taxa of Mt. Oligirtos and 58 of Mt. Farmakas are threatened. A comparison of Mts. Oligirtos and Farmakas with five neighboring mountains shows that elevation correlates positively with the number of regional or bi-regional endemics but not with local or narrow endemics. The importance of mountainous regions for plant conservation is stressed.

## 1. Introduction

The Mediterranean Basin is one of the richest places as far as plant and animal biodiversity is concerned, hosting approximately 25,000 vascular plants, more than half of which are endemic to the area [1]. It has, thus, been placed among the 25 first Global Biodiversity Hotspots [2]. The circum-Mediterranean area, although representing only 1.6% of the Earth’s surface, hosts approximately 10% of the world’s higher plants [3]. Greece, the southernmost part of the Balkan Peninsula, a topographically and ecologically diverse country, hosts at least 7043 native plant species and subspecies (including archaeophytes), 1435 of which are Greek endemics [4,5]. This represents one of the highest degrees of endemism for any comparable area in the whole Mediterranean and Europe [6]. In particular, the floristic (phytogeographical) region of Peloponnisos, as currently defined [4,6], despite its rather limited surface area of approximately 22,140 km^2^ (including surrounding islands), is home to at least 3007 plant species and subspecies [7], 494 of which are Greek endemics [8]. Among them, there are four Greek endemic genera and 77 narrow endemic taxa, i.e., plants with a distribution that does not exceed a linear distance of ca. 50 km [7]. The word *taxa* refers collectively to species and subspecies throughout the article.

Peloponnisos is floristically rather well studied. The first post-Linnean botanist visiting the region was John Sibthorp, who made a trip to Mt. Erimanthos in 1787 and a second, more extensive tour in 1795 [9]. Since then, many botanists have explored Peloponnisos, and the knowledge of its plant diversity has greatly increased. However, there are still many areas that are largely under-investigated floristically, and, therefore, the exact distribution of some endemic plant taxa may not be fully known yet. Many of these under-investigated areas lie on a diagonal line running from NE to SW Peloponnisos [10]. Lying on this diagonal, in the northeastern part of Peloponnisos, the neighboring mountains Oligirtos and Farmakas are to be found, with their flora being the object of the current study.

Very recently, the distribution of the total and Greek endemic flora of Peloponnisos was mapped on the basis of the number of taxa recorded per ca. 10 × 10 km grid cells [8]. The grid cell roughly enclosing Mt. Oligirtos is depicted with 554 taxa at most, while for that enclosing Mt. Farmakas, the number drops to less than 140 taxa. These floristic data are still largely unpublished. When considering endemics, no more than 56 and 22 taxa are shown for the cells containing the two mountains, respectively. In a similar map that appeared ten years earlier [11], the numbers for the Greek endemics in the equivalent cells were less than 26 and 13 for Mts. Oligirtos and Farmakas, respectively. In the same map, the grid cells inscribing the area where Mts. Oligirtos and Farmakas are located have been proposed as important areas for the conservation of Greek endemics (Mt. Farmakas) and Peloponnisos endemics (Mt. Oligirtos) [11].

Endemic plants are important not only as part of the biodiversity under threat during the Anthropocene era and thus in urgent need of protection [12] but also because they directly affect human well-being since many are known to provide important ecosystem services [8]. These plants are unique and often rare and difficult to locate. Their medicinal or aromatic application potentials are largely unknown. Therefore, they act as future resources that may offer exploitation possibilities.

The aims of the current study are: (i) to investigate the vascular plant diversity of Mts. Oligirtos and Farmakas; (ii) to emphasize the endemic taxa of the two mountains, their distribution patterns, pollination modes, and fruit types; (iii) to set conservation priorities for these endemic taxa, according to the IUCN categories of threatened species, their distribution ranges and the vulnerability of their primary habitat, and (iv) to compare the floristic diversity of the Greek endemic taxa amongst the two studied mountains and five neighboring mountains of N Peloponnisos, with respect to their distribution, life forms, habitats, and conservation.

## 2. Results

### 2.1. Floristic Analysis of Mts. Oligirtos and Farmakas

#### 2.1.1. Total Flora

The total flora of Mt. Oligirtos comprises 740 taxa (724 species and 233 subspecies), native, established aliens, and archaeophytes, belonging to 362 genera and 92 families (Table S1). Briefly, 8 of these taxa are *Pteridophyta*, 9 are *Gymnospermae*, and 723 are *Angiospermae*. Out of the 740 taxa, 7 are alien in Greece, established in the area. The flora of Mt. Farmakas comprises 762 taxa (746 species and 235 subspecies), native, established aliens, and archaeophytes, belonging to 376 genera and 92 families (Table S1). Briefly, 8 of these taxa are *Pteridophyta*, 7 are *Gymnospermae*, and 750 are *Angiospermae*. Out of the 762 taxa, 14 are alien in Greece, established in the area. In the whole study area (i.e., both mountains collectively) 996 taxa (967 species and 314 subspecies) have been recorded, belonging to 101 families and 436 genera; 17 of the 996 taxa are established aliens. Families represented with most taxa in the flora of Mt. Oligirtos are *Asteraceae* (86 taxa), *Fabaceae* (69 taxa), *Poaceae* (62 taxa), *Lamiaceae* (45 taxa), *Brassicaceae* (43 taxa), *Caryophyllaceae* (37 taxa), and in that of Mt. Farmakas *Asteraceae* (87 taxa), *Fabaceae* (79 taxa), *Poaceae* (78 taxa), *Lamiaceae* (51 taxa), *Caryophyllaceae* (41 taxa), *Brassicaceae* (38 taxa). For the first time, 635 taxa (616 species and 191 subspecies) are recorded on Mts. Oligirtos and 756 taxa (733 species and 230 subspecies) on Mt. Farmakas, while a total of 878 taxa (870 species and 296 subspecies) have been identified for the first time in the study area. Among these, *Echinops ritro* L. subsp. *ritro*, *Hieracium pannosum* Boiss. subsp. *pannosum*, *Taraxacum copidophylloides* A.J. Richards, *Ononis spinosa* L. subsp. *leiosperma* (Boiss.) Širj., *Ornithogalum kochii* Parl., *Bromus cappadocicus* Boiss. and Balansa subsp. *cappadocicus*, *Poa angustifolia* L., *Rumex thyrsiflorus* Fingerh., and *Prunus spinosa* L. subsp. *spinosa*, as well as the alien *Echinochloa crus-galli* (L.) P. Beauv. subsp. *spiralis* (Vasinger) Tzvelev, are, to the best of our knowledge, reported for the first time in the floristic region of Peloponnisos. Furthermore, the presence of *Salix euxina* I.V. Belyaeva in the same region is hereby confirmed.

#### 2.1.2. Endemic Flora

The Greek endemic flora is represented by 76 taxa (51 species and 33 subspecies) on Mt. Oligirtos and 68 taxa (49 species and 25 subspecies) on Mt. Farmakas. The number of taxa endemic to Peloponnisos found on Mt. Oligirtos is 15 (11 species and 5 subspecies), while on Mt. Farmakas, it is 12 (9 species and 3 subspecies). Among them, *Adonis cyllenea* Boiss., Heldr. and Orph., *Viola oligyrtia* Tiniakou (Mt. Oligirtos), and *Asperula saxicola* Ehrend. (Mt. Farmakas) are narrow endemics. In the study area, 91 Greek endemic taxa (63 species and 36 subspecies) have been recorded, 17 (13 species and 5 subspecies) of which are restricted to Peloponnisos, while 3 species exhibit a very narrow distribution of a linear distance up to 50 km.

Families represented with most Greek endemic taxa in the flora of Mt. Oligirtos are *Asteraceae* (10 taxa), *Lamiaceae* (8 taxa), *Rubiaceae* (7 taxa), *Brassicaceae* (6 taxa), *Boraginaceae* (4 taxa), and *Caryophyllaceae* (4 taxa), and in that of Mt. Farmakas, they are *Asteraceae* (9 taxa), *Lamiaceae* (7 taxa), *Rubiaceae* (7 taxa), *Brassicaceae* (6 taxa), *Caryophyllaceae* (5 taxa), *Boraginaceae* (3 taxa).

**Figure 1:**
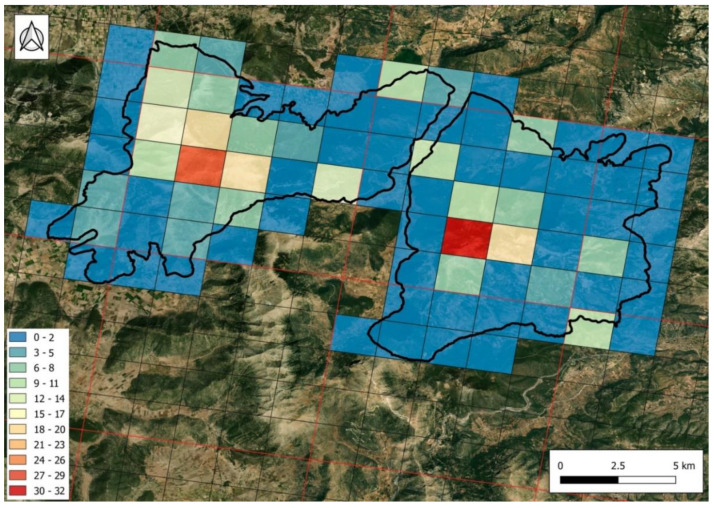
Number of Greek endemic taxa (depicted with color scale) recorded in each of the 2 × 2 km grid cells on Mts. Oligirtos and Farmakas. The study area (black line) and 10 × 10 km European Environment Agency (EEA) grid (red line) are also indicated. Grid cells covered less than 0.2% (i.e., 8000 m^2^) of the study area have been ignored.

After mapping the number of Greek endemic taxa recorded in each of the 2 × 2 km grid cells covering the mountains, it became clear that the highest concentration of endemics is found above the tree line, towards the summits, in both mountains (Figure 1). At lower altitudes, towards the periphery of the mountains, very few grid cells host more than eight endemic plants. In contrast, most grid cells towards the summits host a number larger than 15 (and up to 32) endemic taxa.

Unpublished preliminary data (not presented here) regarding the mapping of European Union habitats, according to the Council Directive 92/43/EEC [13,14], within the study area show that there is a very significant positive correlation between the number of Greek endemics in each grid cell and the area occupied by the habitat types 4090 “Endemic oro-Mediterranean heaths with gorse” (Spearman’s rank correlation coefficient r_s_ = 0.501, *p* < 0.001) and 8210 “Calcareous rocky slopes with chasmophytic vegetation” (r_s_ = 0.611, *p* < 0.001).

#### 2.1.3. Threatened Endemic Taxa and Prioritization

**Figure 2:**
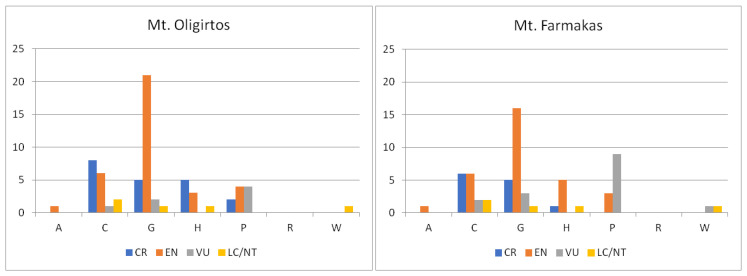
Classification of the Greek endemic taxa of Mts. Oligirtos and Farmakas in IUCN Red List categories, according to the main habitats each of the endemic plants prefers. LC/NT: Least Concerned/Near Threatened, VU: Vulnerable, EN: Endangered, CR: Critically Endangered. A: Freshwater habitats, C: Cliffs, rocks, walls, ravines, boulders, G: Temperate and submediterranean grasslands, H: High mountain vegetation, P: Xeric Mediterranean phrygana and grasslands, R: Agricultural and ruderal habitats, W: Woodlands and scrub. (Kruskal–Wallis one-way ANOVA: H = 16.235, df = 6, *p* = 0.013 for Mt. Oligirtos and H = 15.104, df = 6, *p* = 0.019 for Mt. Farmakas).

For both mountains, there is a statistically significant difference in the distribution of threatened endemic taxa among habitat categories (Figure 2). Most threatened species are found in temperate and submediterranean grasslands, followed by cliffs, rocks, walls, ravines, and boulders. Regarding critically endangered species, cliffs, rocks, walls, ravines, and boulders is the most important habitat category for both mountains, followed by temperate and submediterranean grasslands but also high mountain vegetation for Mt. Oligirtos. Vulnerable taxa seem to be present in many habitats in low numbers, except for xeric Mediterranean phrygana and grasslands on Mt. Farmakas, where their number is higher.

When evaluating the Greek endemic taxa of Mts. Oligirtos and Farmakas by weighing their distribution range according to the endemism categories together with their main habitat vulnerability, seven species and one subspecies have a score higher than ten (Table 1). All of them are endemic to Peloponnisos; the sole exception is the species *Convolvulus mairei*, which is also native to the floristic region of Sterea Ellas. The latter was collected in a single locality on each of the two mountains under study at elevations higher than 1350 m a.s.l. It forms very small, local populations. From the remaining seven taxa, *Adonis cyllenea*, *Viola oligyrtia*, and *Cirsium hypopsilum* were found only on Mt. Oligirtos, while the other four were found on both mountains. *Adonis cyllenea* was collected in a few localities above 1400 m a.s.l. in dolines or small valleys between ridges; *Viola oligyrtia* was also found in just a few localities at elevations of 1100–1250 m, growing in forest openings, forest margins, or in sparse woodland; *Cirsium hypopsilum* was located only in the Goupata plateau at approx. 1500 m a.s.l., in perennial grasslands. All of them form small populations. *Crataegus pycnoloba* was found in small populations in several localities on both mountains, usually above the tree line or in forest margins, and is the most common among the eight taxa discussed here, as reflected also by its IUCN category of threatened species (Least Concerned). *Erysimum pectinatum* was found in one or two localities on each mountain at high elevations (> 1300 m a.s.l.), forming very small populations. *Noccaea graeca* was found in a few localities, forming small populations in open habitats above the elevation of 1150 m. Lastly, the subspecies *Onosma erecta* subsp. *malickyi* was located in a few localities, in open habitats above the elevation of 850 m, but it is locally abundant.

#### 2.1.4. Biological Traits of Endemics

**Figure 3:**
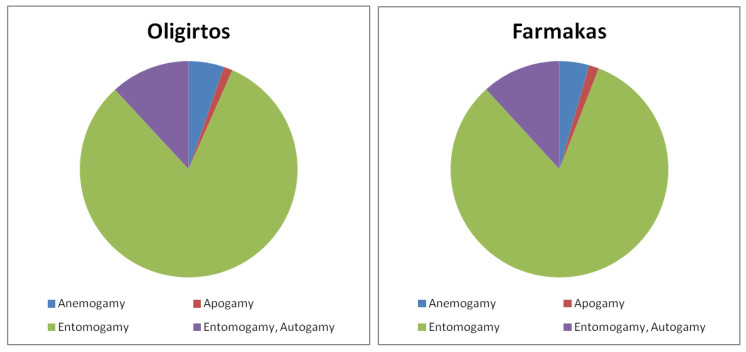
Pollination mode of the Greek endemic taxa found in the flora of Mts. Oligirtos and Farmakas. (Kruskal–Wallis one-way ANOVA: H = 0.19, df = 1, *p* = 0.663).

Regarding pollination, entomogamy is by far the most important pollination mode among the Greek endemic taxa of both Mt. Oligirtos and Mt. Farmakas since 93.4% and 94.1%, respectively, are pollinated by insects (Figure 3). However, 11.8% of the endemic taxa of both mountains are also autogamous. Anemogamous (5.3% and 4.4%, respectively) and apogamous (1.3% and 1.5%, respectively) plants have only a minor contribution to the endemic flora.

**Figure 4:**
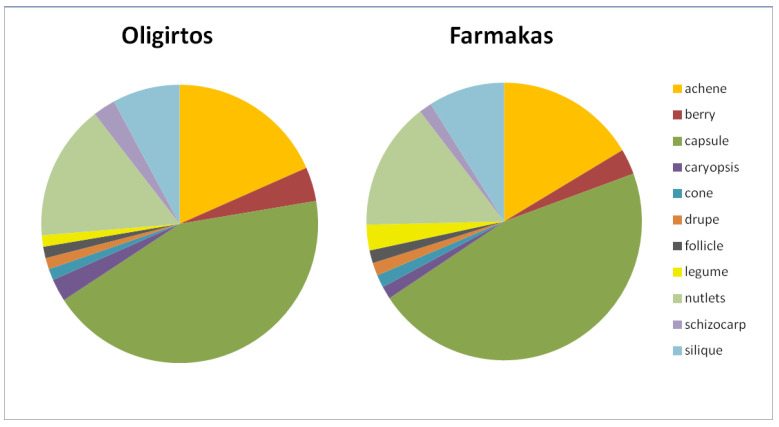
Fruit types of the Greek endemic taxa found in the flora of Mts. Oligirtos and Farmakas. (Kruskal–Wallis one-way ANOVA: H = 0.228, df = 1, *p* = 0.633).

Almost half of the Greek endemic taxa found on Mts. Oligirtos and Farmakas bear capsules, while plants with achenes account for ca. one-sixth of all the endemics (Figure 4). Apart from silique (8% and 9% in the endemic flora of the two mountains, respectively) plants with other fruit types have a minor contribution to the endemic flora.

### 2.2. Comparative Analysis of Endemic Flora among Mountains of N Peloponnisos

#### 2.2.1. Diversity of Endemic Taxa

**Figure 5:**
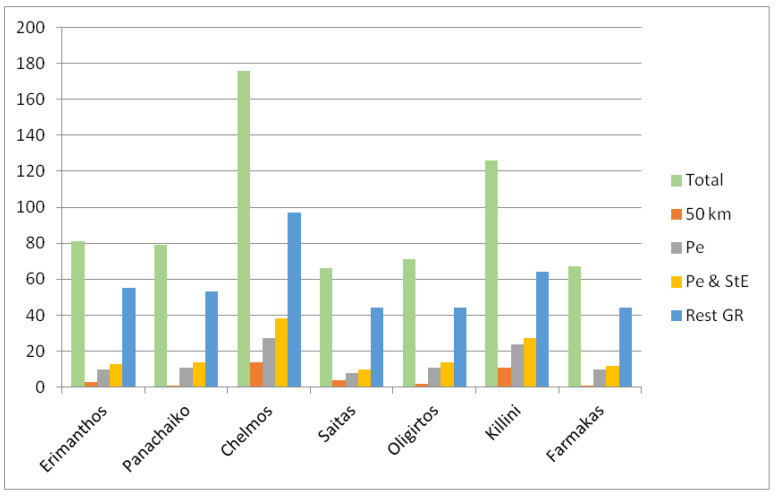
Number of Greek endemic taxa (Total), narrow endemics (50 km), Peloponnisos endemics (Pe), Peloponnisos and Sterea Ellas endemics (Pe and StE), and endemics with a wider distribution in Greece (Rest GR) recorded so far on Mts. Erimanthos, Panachaiko, Chelmos, Saitas, Oligirtos, Killini, and Farmakas. (Kruskal–Wallis one-way ANOVA: H = 5.577, df = 6, *p* = 0.472).

By comparing the Greek endemic flora of the two mountains under study with that of five different, well-studied mountains of north Peloponnisos, namely, Erimanthos, Panachaiko, Chelmos, Saitas, and Killini, it is apparent that the number of Greek endemic taxa (Figure 5, green bars) is strongly and significantly correlated with the maximum elevation of each mountain (Table 2). The same is also true for the endemics of Peloponnisos, Sterea Ellas, and the endemics with a wider distribution in Greece. The distribution of different endemism categories among the seven mountains analyzed (Figure 5, orange, grey, yellow, and blue bars) are very similar and show no statistically significant differences.

The pairwise comparison of the Greek endemic flora of Mts. Oligirtos and Farmakas with five neighboring mountains by means of the Sørensen similarity index reveals their floristic affinities (Table 3). The pairs of Erimanthos–Panachaiko, Chelmos–Killini, Saitas–Oligirtos, and Oligirtos–Farmakas have a rather high floristic affinity (Sørensen similarity index values ≥ 0.7), while Chelmos–Saitas, Chelmos–Oligirtos, and Killini–Farmakas are more distant floristically (Sørensen similarity index values < 0.5). The comparison of all seven mountains by means of the Diserud–Ødegaard multiple-site similarity index reveals a greater similarity among all sites (Diserud–Ødegaard index value 0.77) since higher order similarities are also considered with this index.

#### 2.2.2. Life Forms

**Figure 6:**
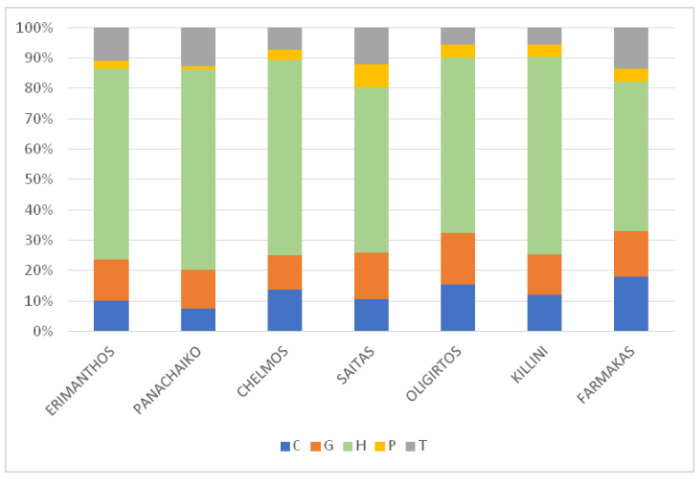
Life form spectra of the Greek endemic flora of Mts. Erimanthos, Panachaiko, Chelmos, Saitas, Oligirtos, Killini, and Farmakas. C: chamaephytes, G: geophytes, H: hemicryptophytes, P: phanerophytes, T: therophytes. (Kruskal–Wallis one-way ANOVA: H = 3.176, df = 6, *p* = 0.787).

Life form spectra of the Greek endemic flora of the seven mountains included in the analyses (Figure 6) reveal that the proportion of each life form category is rather similar among all mountains (Kruskal–Wallis one-way ANOVA: H = 3.176, df = 6, *p* = 0.787). Hemicryptophytes are dominant, their values ranging from 49.3 % (Mt. Farmakas) to 65.8% (Mt. Panachaiko). Phanerophytes, in contrast, have only a minor contribution to the endemic floras.

#### 2.2.3. Habitats and IUCN Categories of Threatened Species

**Figure 7:**
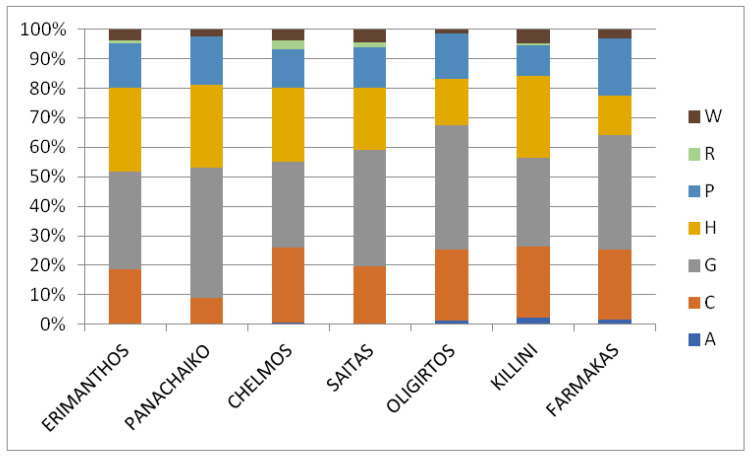
Proportion of the Greek endemic taxa hosted in each habitat category for Mts. Erimanthos, Panachaiko, Chelmos, Saitas, Oligirtos, Killini, and Farmakas. W: Woodlands and scrub, R: Agricultural and ruderal habitats, P: Xeric Mediterranean phrygana and grasslands, H: High mountain vegetation, G: Temperate and submediterranean grasslands, C: Cliffs, rocks, walls, ravines, and boulders, A: Freshwater habitats. (Kruskal–Wallis one-way ANOVA: H = 4.44, df = 6, *p* = 0.617).

The distribution of the Greek endemic taxa of the seven mountains according to the main habitat category each one prefers (Figure 7) reveals that for all seven mountains, the highest numbers of Greek endemics are found in the temperate and submediterranean grasslands, followed by the high mountain vegetation. Two exceptions regard Mt. Farmakas, with xeric Mediterranean phrygana, and grasslands, and Mt. Oligirtos, with cliffs, rocks, walls, ravines, and boulders, which seem to play a more important role in endemic plant distribution than high mountain vegetation. For Mts. Chelmos and Saitas, cliffs and high-altitude vegetation host almost the same number of endemics. Freshwater and ruderal habitats, on the other hand, host a very limited number of endemics in all mountains. However, all the above-mentioned differences are not statistically significant.

**Figure 8:**
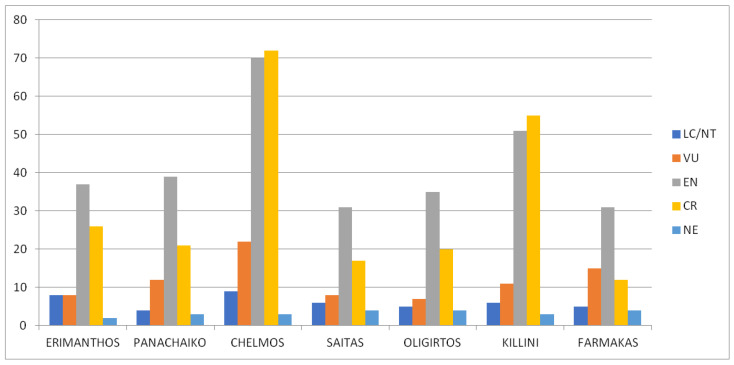
Classification of the Greek endemic taxa of Mts. Erimanthos, Panachaiko, Chelmos, Saitas, Oligirtos, Killini, and Farmakas in the IUCN categories of threatened species. LC/NT: Least Concerned/Near Threatened, VU: Vulnerable, EN: Endangered, CR: Critically Endangered, NE: Not Evaluated. (Kruskal–Wallis one-way ANOVA: H = 1.625, df = 6, *p* = 0.951).

Many of the Greek endemic taxa found on the seven mountains examined are classified in one of the IUCN categories of threatened species, i.e., Vulnerable, Endangered, or Critically Endangered (Figure 8). Most of them fall into the categories “Endangered” and “Critically Endangered”. Mountains like Chelmos or Killini that host a large number of endemic taxa also show high numbers of threatened species.

## 3. Discussion

### 3.1. Floristic Analysis of Mts. Oligirtos and Farmakas

Through the contribution of the current study, it is shown that the total flora of Mts. Oligirtos and Farmakas comprise at least 740 and 762 species and subspecies, of which 76 and 68, respectively, are endemic to Greece. Mt. Farmakas, though ca. 300 m lower than Mt. Oligirtos, hosts a higher plant diversity. This can probably be attributed to the fact that Mt. Farmakas includes areas with a considerably lower elevation than Mt. Oligirtos. These areas are warm and dry and add heterogeneity and higher habitat diversity to Mt. Farmakas. Endemics correspond to 10.22% and 8.89% of the total flora of Mts. Oligirtos and Farmakas. These numbers are comparable to neighboring mountains of similar size and elevation, such as Saitas [15], but remain considerably lower compared to larger and higher mountains, such as Killini or Chelmos [16,17]. The uneven distribution of endemics is also reflected in the Sørensen similarity index values when applied to the endemic floras of the mountains. As a result of our research, the knowledge of the plant diversity of Peloponnisos as a region has been enriched, since 10 taxa (four species and six subspecies) had not been recorded in the area previously, one of them, *Taraxacum copidophylloides* A.J. Richards, being endemic to the country.

#### 3.1.1. Patterns of Endemism

Families with the highest number of taxa in the floras of Mts. Oligirtos and Farmakas coincide to a considerable degree with those comprising most Greek endemics, except for *Fabaceae* and *Poaceae*. The same results have been observed when the endemic flora of the whole country was analyzed: the richest families in the total flora are also represented with the highest numbers of endemics, apart from *Fabaceae* and *Poaceae* which, in spite of being among the three richest and most diverse families in Greece, rank lower in the number of endemics [18]. The families richest in Greek endemic taxa in the study are largely congruent with those represented with most endemics in the flora of Peloponnisos [8] but also nationwide [18]. A similar pattern was observed in the Baetic Mountains of Spain. *Asteraceae* is the richest family in endemics in the area but also in the total Iberian flora. On the other hand, *Fabaceae* and *Poaceae*, which hold the second- and third-ranking positions in the Iberian flora, are less important when it comes to endemics [19].

In Peloponnisos, there is a clear trend for endemics to concentrate in areas with a high elevation range [11]. The same holds true on a smaller scale for Mts. Oligirtos and Farmakas. When the number of endemics per grid cell is mapped, the highest numbers of endemic taxa are clearly located in areas with high elevation. This is further supported by the significant correlation between the number of endemics with the total area covered by the habitat types 4090 and 8210 in each cell. The 4090 habitat type is typical of high, dry mountains and corresponds to the area above the tree line, while the 8210 type refers to the vegetation developed in fissures of limestone cliffs [14]. Both are known to host a large number of endemic species [11,18,20].

#### 3.1.2. Biological Traits of Endemics

The vast majority of the endemics recorded on Mts. Oligirtos and Farmakas are entomogamous. This seems to be the case in other Mediterranean mountainous areas as well as, for instance, in the endemic flora of the Peloritani Mountains in Sicily [21] or in the total flora of the Apennines in Italy [22]. Entomogamy provides high pollination precision, which results in a high percentage of the flowers producing seeds, without the negative genetic effect of increased homozygosity that often occurs as a result of autogamy [23]. At least for some taxa, like for example the *Ophrys* members, there is a species-specific pollination strategy, while most taxa are pollinated by generalist flower visitors.

Almost half of the endemic taxa on the two mountains produce capsules, while a few different fruit types (achenes, nutlets) may also play an important role as widespread fruit structures. Capsules usually contain numerous seeds, and the species may, thus, increase their dispersal and propagation. However, the proportion of different fruit types in the endemic flora should at least partially be attributed to other factors as well. The literature data indicate that *Fabaceae* and *Poaceae* have rather low percentages of endemism in the Greek flora, although they score very high in a number of taxa among the total flora [18]. These findings are also supported but the current study. Legumes and caryopses characterize the fruit types of these two families and because of their low endemicity, not much contribution is expected by these fruit types among the mountain endemics. On the other hand, capsules are produced by a diverse number of species in many different plant families. In the alpine vegetation of the Apennines in Italy, however, achenes are the most common fruit type (46.5%), and capsules follow with 36.21% [22]. This discrepancy can probably be attributed to the much higher elevation of the Apennines as well as the inclusion of the total flora in that study, which is thus not specifically oriented towards the endemics.

#### 3.1.3. Conservation Priorities

In an era where human pressure on ecosystems and biodiversity is growing at a fast pace, conservation has become more important than ever before. The majority of Greek endemics are threatened [12] and thus priorities should be set. The same is true for the mountains of our study. Mt. Oligirtos, known to be a site important for the conservation of Peloponnisos endemics, is home to no less than 62 plant taxa under threat, and Mt. Farmakas, known to be a site important for the conservation of Greek endemics, to 58. As presented, many threatened endemics are found in grasslands, cliffs, high-altitude habitats or phrygana, and annual-rich grasslands. The mountain massifs of Peloponnisos constitute diversity hotspots for threatened Greek endemics [12]. The mountains of southern and central Greece have been included in the 10 Mediterranean Basin hotspots based on plant endemism and richness [3,24]. Moreover, according to recent studies [12], thirteen Greek endemic species and one subspecies are in urgent need of conservation attention since they have a high Evolutionary Distinct and Globally Endangered (EDGE) index, a geographically and threat-weighted variant of phylogenetic diversity that takes into account evolutionary distinctiveness and the IUCN threat category. Three of these species, namely, *Abies cephalonica* Loudon, *Saxifraga sibthorpii* Boiss., and *Rhamnus sibthorpiana* Schult., have been recorded in the study area, and protection measures on Mts. Oligirtos and Farmakas would contribute to their conservation nationwide. Likewise other mountains of the Meditteranean [19], conservation efforts should target on high mountain areas, but medium-high mountain areas should not be disregarded.

### 3.2. Comparative Analysis of Endemic Flora among Mountains of N Peloponnisos

#### 3.2.1. Diversity of Endemic Taxa

The number of Greek endemics found on each of the seven mountains of N Peloponnisos included in our analyses is correlated to the elevation of these mountains. Higher and larger mountains in general host higher plant diversity and species diversity is positively correlated to endemism in the Mediterranean basin [18,21]. The elevation of the mountains also correlates to the number of endemics in Peloponnisos and Sterea Ellas but this relationship does not stand true regarding local Peloponnisos endemics or the narrow endemics. Narrow endemics of Peloponnisos seem to have habitat specialization, and their distribution is more dependent on the patchy distribution of certain habitats rather than elevation itself [11].

#### 3.2.2. Life Forms

While examining the life form spectra of the endemic flora, it becomes obvious that there is little variation among the seven mountains included in our analyses. Hemicryptophytes are dominant in all seven spectra. This is congruent with findings across the country [18] but also with those in other Mediterranean areas. In the Baetic Mountains of Spain, hemicryptophytes dominate in the life form spectrum of the endemic taxa with 45.5% [19]. Therophytes are less represented in the endemic flora of the considerably higher mountains, Killini and Chelmos, and are more prominent in that of Mt. Farmakas, the latter having not only a lower maximum elevation but also comprising areas being hotter and drier. This is to be expected since warmer and drier regions have a higher proportion of therophytes both as a total, as well as in their endemic flora since annual plants are better adapted to arid zones [18]. Similar cases are known from the endemic flora of the Baetic Mountains, where therophytes but also geophytes are found in higher numbers at lower elevations [19], and in the Catalan Pyrenees, where hemicryptophytes dominate in the life form spectrum, although the latter study refers to the total flora and not specifically to the endemics [25]. It is not to be forgotten, however, that all the above-mentioned differences among the seven mountains of N Peloponnisos included in the comparative analysis are not statistically significant.

#### 3.2.3. Habitats and IUCN Categories of Threatened Species

Temperate and submediterranean grasslands is the habitat category invariably preferred by most endemics in all seven mountains examined. For the endemics on Mt. Oligirtos, cliffs and high-altitude vegetation are also of importance. In contrast, for Mt. Farmakas, where subalpine habitats are much reduced in area coverage, cliffs as well as phrygana and annual-rich grasslands are of importance. This is at least partly in compliance with earlier studies [18]. The fact that Mt. Farmakas hosts nine taxa that are predominantly found in high-altitude vegetation, despite its lower elevation compared to the remaining mountains of our study and the spread of *Abies* woodland almost to its summit, is indicative of the utter importance that these particular habitats and the mountains themselves have for the conservation of the endemic flora of Peloponnisos. As far as the IUCN categories of threatened taxa are concerned, many of the endemics fall into these, particularly into the Endangered and Critically Endangered categories. This is also true for the Greek endemics nationwide [12]. Similar findings are known from Spain, where approximately two-thirds of the endemic Baetic Mountain flora is under threat [19]. The fact that the majority of the endemic taxa are hosted in habitats of middle and high elevations, namely, temperate and submeditteranean grasslands, as well as high-altitude vegetation, on all seven mountains examined, supports the importance of mountain massifs in the conservation of Greek endemic plants.

## 4. Materials and Methods

### 4.1. Analyses of Original Data

#### 4.1.1. Study Area

**Figure 9:**
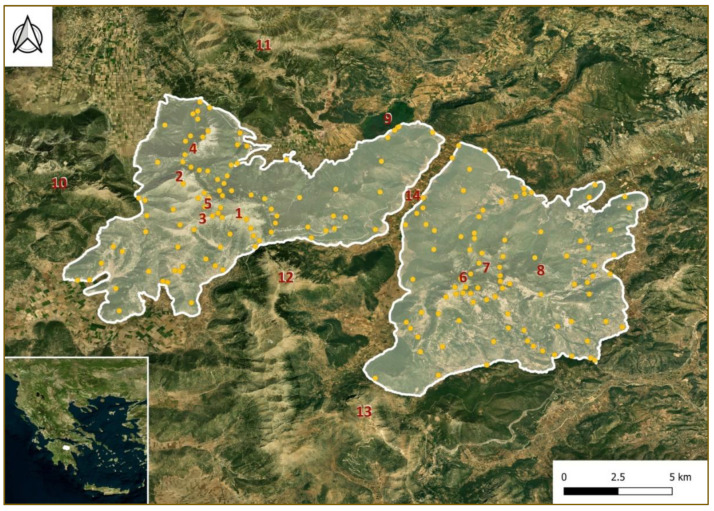
Map of the study area shown in white. Mt. Oligirtos (left) covers a total area of 104.71 km^2^; Mt. Farmakas (right) an area of 114.76 km^2^. Localities, where plant collections were undertaken, are shown by orange dots (neighboring localities in the same habitat have been merged for visualization reasons). The location of the study area in relation to continental Greece is also indicated (insert). 1: Skipiza summit (1935 m), 2: Gribini summit (1831 m), 3: Chionotripa summit (1800 m), 4: Mt. Parnias (1694 m), 5: Goupata plateau (ca. 1500 m), 6: Avizes summit (1615 m), 7: Kastro summit (1510 m), 8: Xerovouni summit (1432 m), 9: Lake Stymphalia, 10: Mt. Saitas, 11: Mt. Killini, 12: Mt. Skiathis, 13: Mt. Lirkio, 14. Xerolakka valley (540 m).

The study area comprises Mts. Oligirtos and Farmakas of NE Peloponnisos, S Greece (longitude 22°17’ to 22°36’ Ε, latitude 37°42’ to 37°52’ N) and covers a total area of 219.47 km^2^ (Mt. Oligirtos 104.71 km^2^; Mt. Farmakas 114.76 km^2^) (Figure 9).

Both mountains were defined either by their lowest elevation, i.e., a col or a brook that separates them from neighboring mountains, or by the point at which they start to elevate when neighboring a plain or a valley. Mt. Oligirtos (Figure 9) is located SSW of Stymphalia lake. It is separated from Mt. Saitas to the west by the col of Profitis Ilias (1080 m a.s.l.) from Mt. Killini to the north by the col of Kastania (1080 m a.s.l.) and from Mt. Skiathis to the south by a col at 1200 m a.s.l. Mt. Farmakas is located to the ENE of Mt. Oligirtos, and the two mountains are separated by a valley called Xerolakka (540 m a.s.l.). At approximately 1500 m a.s.l. on Oligirtos lies a large plateau known as Goupata, surrounded by the highest summits of Skipiza (1935 m a.s.l.), Gribini (1831 m a.s.l.) and Chionotripa (1800 m a.s.l.), as well as a few more, somewhat lower peaks. The altitudinal range of Mt. Oligirtos comprises elevations from 540 to 1935 m a.s.l. The Goupata plateau is rich in karstic phenomena, predominately dolines of various sizes. Dolines are also located higher up towards the peaks. Some karst sinkholes are also present in the same area. The NW part of Mt. Oligirtos, neighboring Mt. Killini, is called Mt. Parnias or Mt. Mavrovouni (1694 m a.s.l.) [26]. The rock formations of Mt. Oligirtos are composed primarily of limestone of two different types and dolomites. Flysch is also found on a smaller scale, while Meso-Pleistocene and Holocene deposits are present at the periphery of the mountain, where it borders valleys [26,27]. The mountain vegetation at low elevations is predominantly composed of evergreen shrubland, replaced by *Abies* forests at higher elevations. Rocky grasslands are prevalent above the tree line. The largest part of the mountain was initially characterized as a Site of Community Importance (SCI) of the Natura 2000 network under the Council Directive 92/43/EEC [13] and later as a Special Area of Conservation (SAC) with the name “Oros Oligyrtos” (GR2530004). Therefore, a detailed study of its flora is crucial for conservation.

Mt. Farmakas is located to the ENE of Mt. Oligirtos (Figure 9). It is separated from Mt. Lirkio to the SW by the col of Agia Kiriaki (900 m a.s.l.). Its highest peaks are Avizes (1615 m a.s.l.), Kastro (1510 m a.s.l.), and Xerovouni (1432 m a.s.l.) [26]. The altitudinal range of Mt. Farmakas comprises elevations from 210 m a.s.l. in the SE to 1615 m a.s.l. A long limestone outcrop stretches from the area SW of Avizes’ summit. The geological background of the mountain is very similar to that of Mt. Oligirtos [26,27]. The physiognomy of its vegetation at low elevations is also composed predominantly of evergreen shrubland, replaced at higher elevations by *Abies* forests that grow almost to the summit.

**Figure 10:**
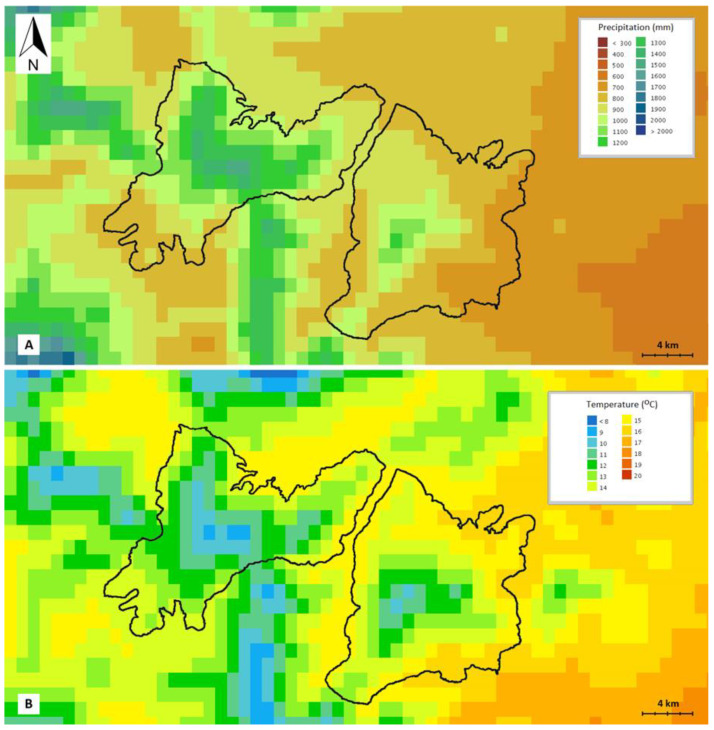
Excerpt of the Climatic Atlas of Greece 1971–2000, according to the Hellenic National Meteorological Service [33]: (**A**) Mean annual precipitation [mm], (**B**) Mean annual temperature [°C]. the Black line indicates the location of Mts. Oligirtos (**left**) and Farmakas (**right**).

The climate of both mountains is Mediterranean, with a dry summer period lasting approx. 4.5 months, from mid-May to the end of September, according to the ombrothermic diagrams [28] based on data retrieved from the site of the Hellenic National Meteorological Service for the meteorological station of Tripoli (reference period 1957–2010) [29]. The bioclimatic state, as defined by Emberger [30,31] and Sauvage [32] falls into the sub-humid climate with cool winters (Q_2_ = 92.59; m = 0.9 °C). However, Mt. Oligirtos, due to its higher elevation and its location to the west of Mt. Farmakas, is colder and more humid than the latter (Figure 10).

#### 4.1.2. Literature Review

A thorough investigation of the floristic literature was undertaken, to obtain an overview of the plant taxa already recorded in the study area. The data were primarily retrieved from [34,35] and to a lesser extent from publications in scientific journals, namely, Phytologia Balcanica [36,37,38,39,40,41,42,43,44,45] or others [46], as well as the volume on the Endemic Plants of the Peloponnese [9]. Moreover, information on herbarium sheets from Mts. Oligirtos and Farmakas were retrieved from the databases of the Herbaria ATH, ATHU, and UPA. A very limited number of entries was found in all three herbaria, and some label data was imprecise, even conflicting. In that case, the collection site could not be included in our study. All the plant species found in these herbaria have also been collected by us in the field.

Floristically, these two mountains have barely been studied to date. In the relevant literature mentioned above, 105 taxa (species and subspecies) are recorded from Mt. Oligirtos and only 6 from Mt. Farmakas. However, some interesting findings regarding rare and/or endemic plants have been recorded on Mt. Oligirtos. Among them, *Biebersteinia orphanidis* Boiss. [47], described from neighboring Mt. Killini in 1854, is the only member of *Biebersteiniaceae* found in Europe, a family otherwise restricted to Asia [48]. It forms small populations on the four mountains of Peloponnisos and shows a disjunct distribution between Peloponnisos and the Taurus Mountains in southern Turkey. It falls into the “Endangered” IUCN category [47]. *Adonis cyllenea* Boiss., Heldr. and Orph., also described from Mt. Killini merely two years later than *B. orphanidis*, is found on the same four mountains [47]. It is a narrow endemic [7] and falls into the “Critically Endangered” IUCN category [12]. For two plants, *Viola oligyrtia* Tiniakou and *Onosma erecta* Sm. subsp. *malickyi* Teppner Mt. Oligirtos serves as the *locus classicus*. The former species, found in *Abies cephalonica* forests, was described in 1991 [47] and later recorded a few kilometers further west on Mt. Saitas [15]. Its IUCN status, according to [12], is “Endangered”. The latter taxon, described in 1988 and growing on boulders and scree at 1100 m a.s.l. [49], is endemic to Peloponnisos [50] and is known from several localities [35,49]. It is characterized as “Critically Endangered” [12].

#### 4.1.3. Data Collection in the Field

In order to assess the floristic diversity of the mountains, 58 daily trips were carried out from December 2016 to May 2022. More than 2400 herbarium sheets were collected, dried, and pressed. Herbarium specimens have been deposited in the Herbarium of the Department of Biology, National and Kapodistrian University of Athens (ATHU). The identification was performed primarily with the aid of the standard Floras for Greece, Europe, and Turkey [6,9,34,35,51,52,53,54,55]. However, some monographs concerning certain plant groups have also been taken into account [49,56,57,58,59,60,61,62,63,64,65,66,67,68,69,70,71,72,73,74,75,76,77]. The material of certain taxonomically difficult genera or groups with unsettled taxonomy (for instance *Ophrys*, *Taraxacum*, *Hieracium*, certain *Poaceae*, etc.) was revised by experts (for details, see acknowledgments).

#### 4.1.4. Nomenclature

The taxonomy and nomenclature follow the online database Flora of Greece Web [50], with the exception of the genera *Salix* [60] and *Ophrys* [57], where more detailed literature has been considered. Wherever possible, the taxa were identified to the level of subspecies. Varieties and hybrids have been omitted. The sole exception regards *Petrorhagia glumacea*/*P. obcordata* (see also Table S1). These two species are not always clearly distinguishable, and hybridization may occur. Material collected during this study may represent hybrids or introgressions.

#### 4.1.5. Chorology, Life Forms, Habitats, and IUCN categories

Chorological categories, life forms, and habitats (Table 4) used for the analyses are according to [4,5], supplemented by data from the online database Flora of Greece Web [50].

The IUCN categories of threatened species for the taxa that have been officially assessed were retrieved from the IUCN Website [78]. However, no formal assessments are available for the majority of the Greek endemic taxa [12]. Thus, the status considered is according to [12] and to a lesser extent according to [11]. For the taxa assigned to more than one category in [12], depending on the criteria used, the highest extinction risk category was considered. Taxa lacking in all the above-mentioned references [11,12,78] were characterized as Not Evaluated (NE). It should be noted that the approach used in [12] to assign endemic taxa to IUCN categories of threatened species “*does not substitute full Red List assessments, but in the absence of such official, time-consuming and resource intensive assessments, it provides a fast, robust and reliable alternative*” [12].

#### 4.1.6. Mapping Hotspots of Endemic Taxa

With the aid of the QGIS software version 3.6.0, a 2 × 2 km grid was established in the study area by dividing each cell of the European Environment Agency (EEA) 10 × 10 km grid into 25 cells. The number of Greek endemic taxa occurring in each 2 × 2 km cell was recorded according to the plant samples collected, possible literature records and our field observations. The number of recorded endemic taxa in each cell is grouped into classes of three (0–2, 3–5, 6–8 taxa, etc.) and mapped using the same GIS software.

#### 4.1.7. Biological Traits of Endemics

The biological traits examined include pollination mode and fruit type (Table S2). The data regarding pollination were partly observed in the field. To this end, the species whose flowers were regularly visited by insects were considered entomogamous while those whose pollen was released by wind breezes were characterized as anemogamous. Our dataset was supplemented by literature findings, primarily the Baseflor database [79], often regarding very similar congeneric taxa, so that a safe conclusion can be drawn. The morphology of certain flower parts also provided useful information: large, feather-like stigmas able of catching pollen are attributed to anemogamous species, whereas the presence of nectaries that offer pollinators a reward is linked to entomogamous plants. In the case of mixed pollination syndromes, the prevailing one was used in our analyses. In order to investigate the reproduction strategy of taxa belonging to the apomictic genera *Pilosella* and *Taraxacum*, the experts on these plant groups, G. Gottschlich (Tübingen) and A. J. Richards (Newcastle), were contacted (in. litt., 2022). Fruit types were retrieved from the herbarium material collected during fieldwork, and also from the descriptions of the species in the standard Floras for Greece, Europe, and Turkey mentioned above.

#### 4.1.8. Prioritization of Plant Taxa

In order to set conservation priorities among the endemic taxa of Mts. Oligirtos and Farmakas, their preferred habitat, according to [4,5,50], as well as the distribution range for each taxon, expressed as one of the above-mentioned endemism categories, were both taken into account. To this end, all habitat categories were given a score, based on the pressures and threats they are prone to, observed during the fieldwork. Moreover, empirical knowledge and literature findings have been considered. The following statements have been evaluated to form the score of the different habitat type categories:

i. freshwater habitats are in general very fragile and threatened by alterations of the water regime, either directly by human intervention or due to effects of global warming and the resulting changes in precipitation patterns [80,81]
ii. grasslands, besides overgrazing that was directly observed in the field, are prone to woody encroachment [82,83,84]
iii. woodlands are prone to wildfires: especially under the pressure of climate change, forests have a higher fire risk [85,86] and thus tend to burn more frequently; furthermore, *Abies cephalonica* Loudon does not possess any active post-fire regeneration mechanism [87]
iv. high-altitude habitats are not particularly threatened, since human activities are rather infrequent in these areas, apart from overgrazing that can pose a threat; however, species adapted to these habitats may not be able to cope with a changing climate [88,89]
v. phrygana are tolerant to disturbance, including grazing and fire, as long as the fire events are not too frequent [90,91,92,93,94]; moreover, phrygana in the study area occur largely due to past human activities, like the abandonment of cultivations and road construction
vi. cliffs are often inaccessible to humans or grazing animals and act as refugia for endemic plants [18,20,95].

For the habitat categories that are found in the study area and host endemic species, a 6-point scoring system was used, with 6 being the most threatened habitat, while for the endemism categories a 4-point scoring system was applied, with 1 covering the widest distribution and 4 the narrowest one (Table 5). Our interpretation is based on the assumption that distribution range affects the extinction risk of plants [78,96]. The two scores are then multiplied, and the taxa are ranked according to their total score value.

#### 4.1.9. Statistical Analyses Software

Spearman’s rank correlation coefficient and Kruskal–Wallis one-way ANOVA statistical analyses were carried out in Statistica 7.0 by StatSoft Inc. (Tulsa, OK, USA).

### 4.2. Comparative Analysis of Endemic Flora among Mountains of N Peloponnisos

In order to compare the Greek endemic flora of Mts. Oligirtos and Farmakas with different mountain regions of northern Peloponnisos, we selected Mts. Erimanthos, Panachaiko, Chelmos, Saitas, and Killini (Table 6). These mountains have been included in the analyses due to their geographical proximity to Mts. Oligirtos and Farmakas and their well-investigated floristic diversity. As the main sources of their plant diversity, we used [15,16,17,97,98] for Mts. Erimanthos, Panachaiko, Chelmos, Saitas, and Killini, respectively, which are the results of long-time studies concerning the flora of the abovementioned massifs. A few more recent floristic records were found in [38,39,40,41,42,43,44,45,99,100,101,102,103,104,105,106,107]. Pairwise similarities among the endemic flora of these mountains were investigated using the Sørensen similarity index [108] while all seven regions were compared collectively through the Diserud–Ødegaard multiple-site similarity index [109].

Greek endemic taxa were divided into four endemism categories, according to their distribution in Greece as reported by [7,50]. These categories include (a) narrow endemic taxa, i.e., plants with a distribution that does not exceed a linear distance of ca. 50 km, (b) Peloponnisos endemics, (c) Peloponnisos and Sterea Ellas endemics, and (d) endemics with a wider distribution in Greece.

The life forms, the preferred habitats, and the IUCN categories of threatened species were compared for the endemic taxa of all seven mountains (Table S3). Nomenclature, chorology, life forms, habitat categories, as well as classification into IUCN categories used in the analyses, follows the same approach as described in 4.1.

Statistical analyses were carried out in Statistica 7.0 by StatSoft Inc. (Spearman’s rank correlation coefficient, Kruskal–Wallis one-way ANOVA) and in Microsoft Office Excel 2007 (Sørensen similarity index, Diserud–Ødegaard multiple-site similarity index).

## Figures and Tables

**Table 1:** Taxa considered highly threatened (total score > 10) due to their narrow distribution and the vulnerability of their main habitat, together with their classification into IUCN Categories of threatened species according to the literature.

Taxon	Total Score	IUCN Category
*Adonis cyllenea* Boiss., Heldr. and Orph.	20	CR
*Viola oligyrtia* Tiniakou	20	EN
*Crataegus pycnoloba* Boiss. and Heldr.	15	LC
*Erysimum pectinatum* Bory and Chaub.	15	EN
*Cirsium hypopsilum* Boiss. and Heldr.	15	CR
*Noccaea graeca* (Jord.) F.K. Mey.	15	CR
*Onosma erecta* Sm. subsp. *malickyi* Teppner	15	CR
*Convolvulus mairei* Halácsy	12	EN

**Table 2:** Results of Spearman’s rank correlation computed to assess the relationship between maximum elevation of Mts. Erimanthos, Panachaiko, Chelmos, Saitas, Oligirtos, Killini, and Farmakas and the number of Greek endemics (Total), narrow endemics (50 km), Peloponnisos endemics (Pe), Peloponnisos and Sterea Ellas endemics (Pe and StE), and endemics with a wider distribution in Greece (Rest GR) recorded on each mountain. r_s_: rho coefficient, *p*: probability.

Endemism Category	r_s_	*p*
Total	0.8929	0.01
50 km	0.7208	0.07
Pe	0.7456	0.05
Pe and StE	0.8108	0.03
Rest GR	0.8524	0.01

**Table 3:** Pairwise comparison of the Greek endemic flora of Mts. Erimanthos, Panachaiko, Chelmos, Saitas, Oligirtos, Killini, and Farmakas by means of the Sørensen similarity index.

	Panachaiko	Chelmos	Saitas	Oligirtos	Killini	Farmakas
Erimanthos	0.75	0.54	0.63	0.57	0.57	0.58
Panachaiko		0.49	0.57	0.55	0.49	0.55
Chelmos			0.47	0.46	0.70	0.43
Saitas				0.73	0.59	0.66
Oligirtos					0.52	0.75
Killini						0.49

**Table 4:** Habitat categories used in the analyses, as described in [4]. Coastal habitats (M) were not found in the study area.

Habitat Abbreviation	Habitat Category Descriptor
A	Freshwater habitats (aquatic habitats, springs and fens, reedbeds and damp tall herb vegetation, seasonally flooded depressions, damp and seepage meadows, streambanks, river and lake shores)
C	Cliffs, rocks, walls, ravines, boulders
G	Temperate and submediterranean grasslands (lowland to montane dry and mesic meadows and pastures, rock outcrops and stony ground, grassy non-ruderal verges and forest edges)
H	High mountain vegetation (subalpine and alpine grasslands, screes and rocks, scrub above the tree line)
M	Coastal habitats (marine waters and mudflats, salt marshes, sand dunes, littoral rocks, halo-nitrophilous scrub)
P	Xeric Mediterranean phrygana and grasslands (Mediterranean dwarf shrub formations, annual-rich pastures, and lowland screes)
R	Agricultural and ruderal habitats (fields, gardens and plantations, roadsides and trampled sites, frequently disturbed and pioneer habitats)
W	Woodlands and scrub (broadleaved and coniferous forest, riparian and mountain forest and scrub, hedges, shady woodland margins)

**Table 5:** Score assigned to habitat and endemism categories. Abbreviations for habitat categories as in Table 4, endemism categories as in Table 2. Coastal, as well as ruderal and agricultural habitat categories (listed as M and R in Table 4), have been omitted since the former category does not occur in Mts. Oligirtos and Famrakas, and the latter does not host any Greek endemic species in the same area.

**Habitat Category**	**Score**
A	6
G	5
W	4
H	3
P	2
C	1
**Endemism Category**	**Score**
50 km	4
Pe	3
Pe and StE	2
Rest GR	1

**Table 6:** Mountains included in comparative analyses of endemic plant taxa.

Mountain	Highest Peak [m a.s.l.]	Lowest Elevation Approx. [m a.s.l.]	Elevation Range Approx. [m]	Area Approx. [km^2^]	Main Geological Substrates *	Latitudinal Range	Longitudinal Range	Taxa (nr. of sp. and subsp.)
Erimanthos	2223	400	1800	510	L, F, C	37°51’ to 38°04’ N	21°43’ to 22°01’ E	979
Panachaiko	1924	200	1700	177	L, F, Sc, C	38°07’ to 38°17’ N	21°47’ to 21°57’ E	829
Chelmos	2355	25	2300	655	L, D, Sa, C, P	37°47’ to 38°11’ N	21°57’ to 22°19’ E	1478
Saitas	1812	450	1400	127	L, D, F, Sa	37°43’ to 37°52’ N	22°08’ to 22°20’ E	800
Oligirtos	1935	540	1400	105	L, D, F	37°44’ to 37°52’ N	22°17’ to 22°29’ Ε	740
Killini	2375	200	2200	233	L, D, Sa, C	37°53’ to 38°03’ N	22°21’ to 22°31’ E	1021
Farmakas	1615	210	1400	115	L, D, C	37°42’ to 37°50’ N	22°27’ to 22°36’ Ε	762

* C: conglomerate, D: dolomite, F: flysch, L: limestone, P: phyllite, Sa: Sandstone, Sc: Schist.

## Data Availability

Not applicable.

## Supplementary Materials

The following supporting information can be downloaded at: https://www.mdpi.com/article/10.3390/plants11192649/s1, Table S1. List of vascular plant taxa identified in the study area of Mts. Oligirtos and Farmakas. Chorological categories according to [4,5,50]. In the *Chorology* column, the origin of the alien taxa is given in square brackets []; * indicates plants that are endemic to Greece at the subspecies level, but not at the species level. Ol: Mt. Oligirtos, F: Mt. Farmakas, Pe: Floristic region of Peloponnisos. Table S2. Endemic plants of Mts. Oligirtos and Farmakas and their biological traits examined. Table S3. Endemic plants of Mts. Erimanthos, Panachaiko, Chelmos, Saitas, Oligirtos, Killini, and Farmakas and their IUCN categories, endemism categories, life forms, and habitats used in the comparative analyses. For abbreviations and sources of the data see the text. Reference [110] are cited in the supplementary materials.
